# Let's Be Straight Up about the Alcohol Industry

**DOI:** 10.1371/journal.pmed.1001041

**Published:** 2011-05-31

**Authors:** The PLoS Medicine Editors

## Abstract

In this month's editorial, the *PLoS Medicine* Editors call for more attention to the practices and influence of the alcohol industry on health research, government policy, and public perceptions.

If medical journals and public health advocates are concerned with corporate conflicts of interest, inappropriate marketing to children, impotent self-regulation, and general flouting of the rules, why are we ignoring the alcohol industry?

The crisis of confidence that surrounds the behavior and practices of Big Tobacco and Big Pharma [1],[2]—bias in funded research, unsupported claims of benefit, and inappropriate promotion and marketing, among others—should be enough to provoke in us all a high degree of skepticism with any industry involvement in health research and policy. But the evidence and critical voices highlighting the practices of the alcohol industry—a massive and growing US$150 billion global business—have not yet received adequate prominence in medical journals. Indeed, attention to and scientific research on the alcohol industry have not kept pace with the industry's ability to grow and evolve its markets and influence in the health arena [3].

So why are we soft on alcohol? One reason might be the enduring perception that drinking is normal, fun, and healthy, and that the damage caused by alcohol affects only a small group of people who can't handle their booze [4]. But the independent statistics defy this rosy view: the Global Burden of Disease study places alcohol-related morbidity second only to tobacco in the developed world [5], teenage drinking problems have been shown to have long term effects on individuals and communities [6], and a recent European-wide study [7] found that 10% of cancers in men and 3% in women were linked to alcohol consumption.

While the statistics on alcohol's harms are troubling enough, it's the practices of the alcohol industry, including its influence on government policy, health research, and public perceptions, that really begs for more of our attention. Several recent examples signal a need for more scrutiny.

In the UK, there have been scathing allegations [8] that the current government is too close to the drinks industry, including its recent invitations allowing industry representatives to influence public health policy, which led to a withdrawal of support for a key alcohol policy by major organizations including the British Medical Association, Royal College of Physicians, and several alcohol control charities [9],[10]. Similar interference in government policy by the alcohol industry, in which scientific evidence was ignored and industry interests inserted into national alcohol policies, was recently documented for sub-Saharan Africa [11].

In the US, a recent review [12] of alcohol industry–funded health research found very little that could contribute to reducing alcohol-related illness. But, worryingly, Barbor did find a lot of potential for the alcohol industry's involvement in science—whether supporting individual scientists, research councils, conferences, or journals—to result in messages that obscure public perceptions of the true benefits and harms of alcohol and to support the industry's PR agenda, while also supplying industry with the opportunity to “demonstrate corporate responsibility in its attempts to avoid taxation and regulation” [12].

Recent analyses have also shown the alcohol industry's savvy in deflecting government controls aimed at protecting the public—for example, the industry's marketing innovations in the use of social media, sports sponsorships, and product placements in film are said to be designed to evade policies restricting broadcast and print ads [13]. And, Hastings and colleagues [14] last month demonstrated how UK alcohol companies and their PR firms continue to market to youth, encourage drunkenness, and link drinking to sociability and social success despite explicit self-regulatory codes prohibiting this type of advertising.

None of this would surprise the Marin Institute (http://www.marininstitute.org/site/), the California-based alcohol industry watchdog, whose work has documented a laundry list of misdeeds by “Big Alcohol”: promoting the health benefits of alcohol while downplaying harms; deflecting attention away from scientific data that contradict industry exaggerations of benefit; tactically targeting specific markets of underage youth, people of color, and poor people; and engaging in philanthropy to promote brand loyalty.

If this questionable behavior is reminiscent of the strategies developed by the pharmaceutical, tobacco, and other industries to further their agendas, it should be a wake-up call to us all. And, as with the pharmaceutical and tobacco industries (whose marketing budgets far exceed the public funding of independent research), efforts to counter such dubious tactics face a formidable and well-resourced industrial opponent. However, experience with other industries, especially through tobacco control efforts, can also teach us a lot about how to critically examine and resist the alcohol industry's behavior and practices. Galvanizing the support of non-governmental organizations and governments, along with a solid base of independent evidence, led to the ratification of the Framework Convention on Tobacco Control, and there have been proposals for a similar Framework Convention on Alcohol Control [15],[16]—a move that would recognize the need for collective global action and could counter the alcohol industry's age-old attempts to individualize responsibility for problem drinking and deflect attention away from their own role in promotion.

Whether the solutions are stricter regulation over advertising and promotion, banning sports sponsorships, setting minimum pricing, restricting access, introducing mandatory safety labeling, or holding the industry to account for the harms associated with their products, there is a need now to target more attention to and research on the alcohol industry that can support and fuel legislative, regulatory, and community action to protect the public health. Let's be straight up about the alcohol industry.
